# Impact of Virtual Reality Cognitive and Motor Exercises on Brain Health

**DOI:** 10.3390/ijerph20054150

**Published:** 2023-02-25

**Authors:** Beata Sokołowska

**Affiliations:** Bioinformatics Laboratory, Mossakowski Medical Research Institute, Polish Academy of Sciences, 02-106 Warsaw, Poland; beta.sokolowska@imdik.pan.pl

**Keywords:** cognitive and motor functions, brain, brain health, brain disorders, exercise, training, virtual reality

## Abstract

Innovative technologies of the 21st century have an extremely significant impact on all activities of modern humans. Among them, virtual reality (VR) offers great opportunities for scientific research and public health. The results of research to date both demonstrate the beneficial effects of using virtual worlds, and indicate undesirable effects on bodily functions. This review presents interesting recent findings related to training/exercise in virtual environments and its impact on cognitive and motor functions. It also highlights the importance of VR as an effective tool for assessing and diagnosing these functions both in research and modern medical practice. The findings point to the enormous future potential of these rapidly developing innovative technologies. Of particular importance are applications of virtual reality in basic and clinical neuroscience.

## 1. Introduction

The global internet (Internet of Things, IoT) and innovative information and communication technologies (IT/ICT) are already ubiquitous in the social and individual lives of people [1,2,3,4,5,6]. Among them, a very significant role is played by Extended Reality (XR) and its classic components such as Virtual (VR), Augmented (AR) and Mixed (MR) Reality [7,8,9,10,11]. There are many indications that these modern technologies (XR, i.e., VR/AR/MR, and 3D printing/scanning, holography, artificial intelligence and machine learning, robotics or online (VR) tele-medicine) will be the basis and determinants of the organization and functioning of contemporary and future generations [12,13,14,15,16,17]. Most likely, they will increasingly affect everyday life, culture, advertisement, business, military, sports, education, science and medicine [18,19,20,21,22,23,24].

This is because these technologies inspire ideas and the possible development of contemporary advanced algorithmic sciences, models of computation, data mining or big data science, and modern (bio) (medical) (neuro) information and communication technologies tools [25,26,27,28]. The findings of neuroscience research focus on the nervous system, especially the brain, its structure and functions, and its importance in both health and disease, simply enabling the implementation of IT/ICT in, for example, sports, education, or basic/clinical neuroscience and computation medicine [29,30,31,32,33,34,35]. On the other hand, these innovative technologies are becoming an increasingly important element in creating new directions of research, from neurobiology, neurogenetics, neurophysiology, neuropsychology, neuropsychiatry, neuropedagogy, and neurogeriatry, through neurotherapy and neurorehabilitation, to (bio)neuroinformatics [36,37,38,39,40,41].

In this context, an interesting issue is the use of innovative technologies in brain research, especially the impact of virtual training/exercise on brain health, specifically, the application of virtual tools in neuroscience to assess and understand the functioning of both the nervous system and other bodily systems in health and disease. One of the major benefits of VR is that it provides an immersive and more naturalistic environment that can increase the ecological validity of interventions or experiments. Moreover, the implementation of virtual reality opens up a wide range of possibilities for the development of dexterity, speed and precision of movement. In addition, it appears to allow for overcoming, especially for practical training, some of the limitations of real exercises [42,43,44,45,46]. Some studies in virtual worlds demonstrate that these environments have high ecological value, as well as high sensitivity and selectivity, for example in modern medical diagnostics [47,48,49]. They are also a great hope for modern virtual (neuro) therapy, (neuro) rehabilitation and health-promoting prevention in (neuro) geriatrics [50,51,52,53,54,55,56,57].

This review presents some of the interesting and promising research on these topics, from popular systems with non-immersive environments (such as the virtual platform used in our lab) to full immersion systems. These systems require the fulfillment of certain criteria, indicated, for example, by the National Academy of Neuropsychology and the American Academy of Clinical Neuropsychology, for the implementation of innovative digital technologies for research and clinical purposes. The literature is large, and the number of publications is growing very rapidly from year to year, so the review is necessarily the subjective view of the author, and also based on her own and similar research in virtual worlds.

## 2. Examples of Virtual Environments (VR, AR and MR) and Their Applications

**Figure 1:**
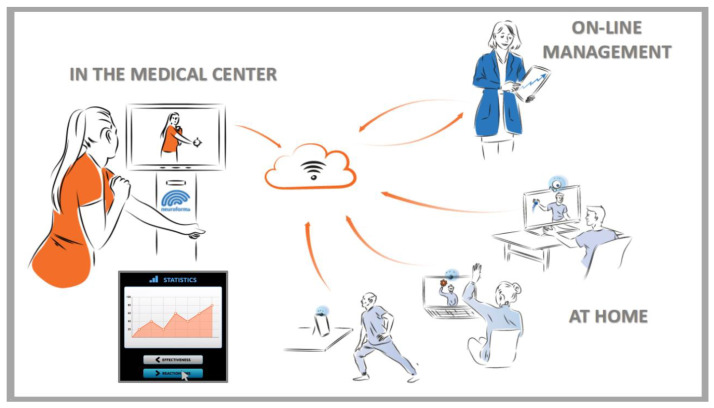
The illustration of basic applications of innovative IT/ICT technologies, e.g., for the VR NEUROFORMA system, which is used in our laboratory: use in stationary settings such as research/medical centers or hospitals, remotely via mobile devices such as iPhones and tables, or using PCs at home (materials from our lab repository, with permission of the Titanis Ltd., Worcester, UK).

The development of innovative IT/ICT technologies and their applications in many areas of modern lives have greatly accelerated with the COVID-19 pandemic. This has been reflected in the development of various computer systems using virtual reality technologies. The virtual systems on offer allow their use both in centers or facilities (e.g., educational, sports, cultural, tourist, military, scientific, medical, etc.) and at home (as shown in Figure 1, for the multi-tasks VR system that is used in our laboratory [58]).

In 2009, the International Society for Virtual Rehabilitation (ISVR) was founded, which not only initiates scientific and research activities in the field of virtual (tele) rehabilitation but also fosters the exchange of information and enhances cooperation between the medical and scientific community and specialists from other, non-medical fields [59]. In general, virtual worlds are still an unknown area of human activity, with an unpredictable impact on human health. Table 1 presents the main applications of virtual environments. Currently, many studies confirm the usability and ecological validity of virtual environments, situations or tasks, mainly for evaluating cognitive processes such as executive functions, memory, visuospatial analysis, and daily functioning. For example, in the neuropsychological diagnosis of executive functions (EFs) cognitive flexibility or planning is often assessed, and in memory processes working and episodic memory and attention skills are examined. The EFs research is important because executive dysfunctions constitute a significant public health problem: their high impact on everyday life makes it a priority to identify early strategies for evaluating and rehabilitating, e.g., brain disorders in a real-world context. The ecological limitations of traditional neuropsychological testing and some difficulties in performing tests or training in real-life scenarios have paved the way for the use of virtual reality-based tools for the assessment and rehabilitation of EFs. VR tests are often based on tasks from everyday life, such as behavior in the classroom, kitchen, supermarket, park, street, city or while driving, etc. Most of these tests are designed to assess EFs using the patterns of real tasks.

For example (see Table 1), the popular VR system known as the Virtual Classroom (VC) task/test is a modern alternative form of cognitive diagnosis when examining children with attention-deficit hyperactivity disorder (ADHD) [63]. The Virtual Action Planning-Supermarket (VAP-S) test by Klinger and co-workers is used to assess various executive functions in several patient groups, such as those with stroke or schizophrenia [64]. Similarly, the Virtual Reality Kitchen proposal by Allain and colleagues [61] demonstrates the important relationship between virtual and real coffee brewing environments, thus supporting the ecological validity of the non-immersive Virtual Coffee Task (NI-VCT). AD patients performed worse on the NI-VCT than healthy elderly controls. Moreover, the results show that VR tests detect subtle deficits (in patients with MCI, AD and PD) that may be unnoticed using traditional assessment methods. Porffy’s group [75] proposes an interesting advanced analytical construct of model validity of age-related cognitive decline (healthy individuals) using a novel VR shopping task. Another interesting VRLAT test (Virtual Reality Lateralized Attention Test), by Buxbaum and colleagues, also aims to effectively assess cognitive and motor functions in both healthy control and patients with unilateral spatial neglect (USN) or Alzheimer’s-type dementia [60]. Zając-Lamparska’s team shows positive effects of VR-based cognitive training of the GRADYS system in older adults without and with middle dementia [65]. A study by Brachman and colleagues [70] presents an interesting innovative VR system and the positive effects of virtual training on the elderly in gait, balance, and cognitive functions. Additionally, motor-cognitive dual tasking in VR games provides additional challenges and supports motor learning processes. Rutkowski and co-workers [71] show that a VR system with immersive music games has the potential to improve hand-eye coordination and reaction time in young musicians. Lorenzis’ team [74] proposes interesting new research on procedural training in relation to the learning path, and compares traditional learning and learning-by-teaching approaches (TL versus LBT). These experimental results demonstrate that the use of Virtual Reality Training Systems (VRTS) alone improved the participants’ performance compared to traditional experiences, and that lecture-based and/or lab-based teaching (LBT) proved more effective than traditional learning (TL). Another clinical study carried out by Stryla and Banas [62] showed beneficial therapeutic effects in post-stroke patients examined with the non-immersive VR NEUROFORMA system. The presented studies were conducted in various virtual environments, from non-immersive to immersive systems, and the examination time was not long. These VR systems are still being developed and upgraded; for example, the VRLAT has been prepared in successive versions for iPhone and CAVE [76].

The resulting various VR environments/situations, such as virtual cities, school classes, kitchens or supermarkets, have been developed for functional assessment and treatment/rehabilitation outcomes. Many clinical results show that, when difficulties occurred in virtual tasks/exercises, they were also observed in real tasks. However, the standards and safety of using of digital technologies are still lacking. In the preparation of recommendations and norms, research with healthy people as well as with patient groups in VR is very valid in order to create effective, safe and attractive exercises and training that will later be implemented in practice. With this approach, it is, additionally, possible to continue the activity of users at home, under the supervision and remote control of a trainer or therapist, with the possibility of modifying such virtual home sessions (Figure 1).

## 3. Basic Features, Importance and Possibilities of Virtual Exercise and Training

Modern fast-developed game-based virtual reality technologies offer many VR environments as a very interesting and attractive form of serious games; this applies to both cognitive tasks and the physical engagement of participants in such VR worlds, for example the VR environments in Table 1. The difference between these professional systems and other games of this type is that the exerciser’s activity is not random, but designed and adapted to the individual’s abilities or needs, and in addition the user and the researcher or therapist can control and even modify the individual (personal) training, and track the VR scores on/offline. In addition, databases of tasks and exercises are prepared and tested in collaboration with various specialists and experts, therapists and neuroscientists [59], including the consideration of the latest finding in neuroscience, such as those on (meta) neuroplasticity, brain asymmetry or mirror neurons phenomena [77,78,79,80,81,82,83,84].

**Figure 2:**
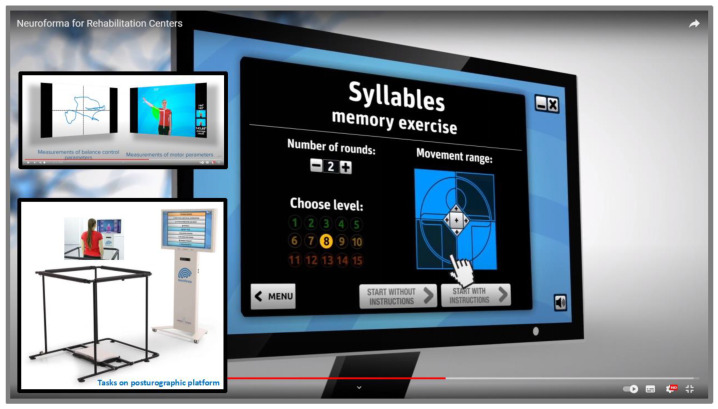
The view of the menu of the Syllables exercise parameters, regarding the selection of the number of rounds, level of difficulty, and range of movement in the VR NEUROFORMA system, and also the view of its version with the posturography platform and safety barriers (materials from our lab repository, with permission of Titanis Ltd.).

Computer systems with virtual environments enable the graded difficulty and differentiation of individual tasks, which allows better use of cognitive-motor learning strategies [85,86,87]. Hence, researchers even suggest that these IT/ICT technologies have advantages over the conventional models of improvement in implementing motor learning principles and applying sensory biofeedback [88,89,90,91]. Figure 2 presents an example of a menu for selecting the parameters of the Syllables exercise, such as the number of rounds (repetitions), the difficulty level of the task, and the range of movement. Based on the scores obtained by the trainees, the relevant statistics of the measurement parameters are prepared, as well as on/offline partial and summary reports. It is also possible to record actual sessions, and then all materials can be used for further in-depth mathematical and statistical analysis and evaluation of the results and effects obtained. Subsequent degrees of task difficulty involve, among other things, additional cognitive and/or motor elements, and modification of the exercise/test conditions, as well as the addition of distracting elements (distractors). The presence of distractors is significant because it requires greater concentration on the task. For example, studies by Negut’s or Buxbaum’s teams have shown the negative impact of visual and auditory distractors on attentional performance in children with ADHD in the VC test [63], and USN patients in the VRLAT [60] or AD patients in the VR coffee task [61]. Similarly, in our modeling studies with healthy adults [68,69,92,93], we observed a decrease in performance at the highest difficulty levels of exercise with additional elements such as various distractions or other parameters of training sessions (such as shorter exposure/decision time, the greater number of VR objects or commands/tasks).

Based on the results of a number of neuroscientific studies, researchers and clinicians point out that diagnostic tests using the virtual reality environment have high accuracy, sensitivity and specificity in identifying various dysfunctions and/or deficits in examining subjects with serious illnesses such as neurodevelopmental disorders (e.g., attention-deficit hyperactivity disorder), schizophrenia spectrum (e.g., schizophrenia), mood (e.g., depressive disorders), anxiety (e.g., panic and phobias), trauma- and stressor-related disorders (e.g., post-traumatic stress disorder), neurocognitive (e.g., Alzheimer’s and memory cognitive impairment diseases) and neuromuscular disorders (e.g., multiple sclerosis, amyotrophic lateral sclerosis). Moreover, VR environments can be not only a diagnostics tool, but also an effective (neuro) therapeutic method. In addition, VR systems are increasingly being incorporated into the study of natural aging processes and the estimation of effective support in (neuro) geriatric care (e.g., effective cognitive intervention or prevention of falls in the elderly).

For example, the research group of Msika and co-workers [72], in an interesting study involving healthy young and older adults, proposed using the non-immersive VR REALSoCog task to assess social cognition in normal aging. A total of 27 situations were selected to assess the participants’ social cognitive processes: 11 control/neutral situations and 16 experimental ones. The experimental situations were specifically developed to investigate social norms by displaying conventional and moral transgressions. The researchers found that the REALsoCog task enabled the assessment of several socio-cognitive processes, such as the ability to make moral and conventional judgments, emotional empathy, affective and cognitive ToM (theory of mind), and the propensity to act in a socially appropriate or inappropriate way. The authors noted that the traditional socio-cognitive tasks can evaluate these processes separately and lack ecological validity, and showed that the new virtual approach is an interesting integrative measure of socio-cognitive functioning that better reflects social behavior in daily living.

Equally interesting research is being conducted by the Kourtesis group [66,67] in the immersive environment of the Virtual Reality Everyday Assessment Lab (VR-EAL) using the HTC Vive HMD, which simulates tasks similar to real life to assess prospective memory, episodic memory, executive functions, and selective visual, visuospatial and auditory attention. It is noteworthy that all VR-EAL tasks significantly correlated with relevant ecologically justified paper-and-pencil tests; therefore, performing the VR tasks can be a predictor of the true real-world functioning of individuals. Moreover, the results showed that age and education did not correlate with scores on VR-EAL and paper-and-pencil tests (participants aged 18–45) [66]. The gaming experience was strongly and positively associated with the VR experience, with gamers completing the assessment faster than non-gamers, not only in VR tasks but also in the paper-and-pencil tests. The researchers reported that the VR-EAL is the first immersive VR neuropsychological battery with increased ecological validity for the assessment of everyday cognitive functions, offering an enjoyable testing experience without inducing cybersickness. The authors emphasized that the VR-EAL meets the criteria of the National Academy of Neuropsychology (NAN) and American Academy of Clinical Neuropsychology (AACN), solves some methodological problems and provides benefits in neuropsychological testing. The studies demonstrate that the VR-EAL can be an effective, reliable and ecologically valid tool for assessing (everyday) cognitive function in both research and clinical practice.

In turn, Aubin and colleagues [64] examined how virtual scores of VAP-S relate to the scores of a shopping task in a real-life supermarket. To use VAP-S, participants sat or stood in front of a computer screen and interacted with the virtual environment using a mouse and keyboard. The screen showed instructions and items to buy. During the shopping task, an error was automatically recorded if the participant tried to add an item to the shopping cart that was not on the shopping list or tried to make a purchase more than once. In the next step, the user had to go to checkout and make a payment by clicking on the wallet icon. If the subject did not pay for these purchases, an error was recorded in the test report. The task was completed when the participant left the supermarket with the shopping cart. The authors found that the scores in both environments were generally similar based on the scores of the observational (classical) assessment tool. The researchers noted that when subjects had difficulty with cognitive strategy in the VR supermarket, they also had similar difficulties in the ecological/naturalistic supermarket.

Another team, Zając-Lamparska and colleagues [65], presented the GRANDYS game with the Oculus Rift DK2 and Vbox 6DOF control pad in healthy and MCI older adults. The GRANDYS system is VR-based cognitive training with four modules: attention, memory, language and visuospatial processing. The storyline of each module scenario consists of tasks inspired by daily life. Each module has three levels of difficulty. The game software included a tutorial module to help participants learn how to operate the game interface. In each module, the participants started at the lowest difficulty level and moved up to a higher level in the next session once they had reached 75% accuracy in the previous one. If accuracy fell below 50%, participants returned to a lower difficulty level. Subjects were accompanied by a training assistant throughout the session. This study showed training progress in older adults, larger in the healthy control. Improvements were observed in visuospatial processing, visual aspects of memory and working memory.

The next proposal of the Virtual Reality Lateralized Attention Test (VRLAT) from Buxbaum’s group [60] required participants to navigate a virtual, unbranched path, either propelling themselves using a joystick (the participant condition) or passively viewing the surroundings while the examiner navigated the path at a constant speed (the examiner condition). Participants were asked to identify virtual objects on either side of the path and avoid collisions with them. The software signals collisions with virtual objects, with auditory feedback corresponding to obstructed progress. The VRLAT includes three array conditions (simple, complex, and enhanced), each with 20 target objects (10 on each side of the path). The scoring system adapted in VRLAT awarded points based on the quality of the answers given, e.g., one point for items named specifically and correctly and zero points for any other answer. The maximum possible score for each side at each level would therefore be 20 points (a 20-point scale). These researchers emphasized that patients with a wide range of cognitive and physical ability levels could perform the VRLAT. The authors pointed out that the VRLAT is an easy-to-administer, computerized task that can be run on any standard personal computer without the need for specialized equipment. Moreover, it is not only a diagnostics method but also a useful assessment tool in studies evaluating response to treatment or natural recovery processes after central nervous system injures.

**Figure 3:**
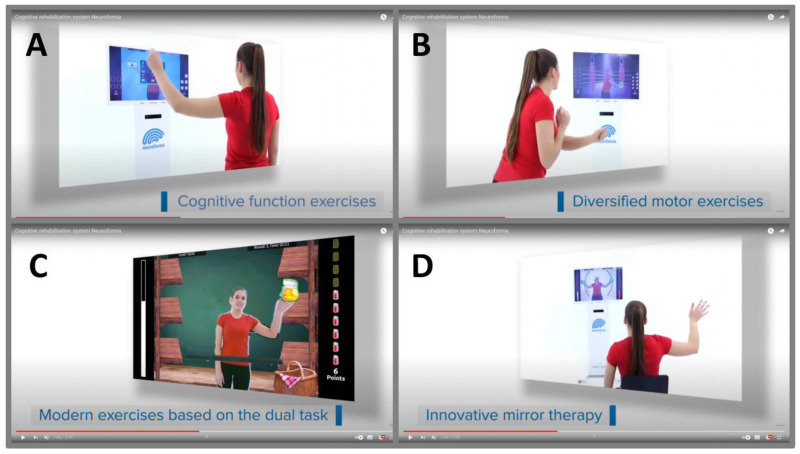
The presentation of examples of various exercises in the VR NEUROFORMA environment, such as (**A**) the arithmetic operations executive exercise, (**B**) the opposing boxing motor exercise, (**C**) the market products cognitive-motor exercise based on the dual task, (**D**) mirror paths exercise (materials from our lab repository, with the permission of Titanis Ltd.).

Our modeling research focuses on functional brain asymmetry in VR NEUROFORMA [68,69,92,93]. After an initial phase of exercises and learning tasks in this system, healthy subjects practiced the virtual test training sessions at least twice on both hands, the right and left hand, and the same exercises were performed in the full, right and left ranges of motion on a posturographic platform (in random order for each subject). Participants stood in front of a screen with a 3D Kinect camera and saw themselves mirrored among VR objects, and their task was to catch, avoid, track and hit these visuals. The algorithm system awards points for the task performance, estimating scoring distribution as a very good performance in the range of 95–100 points and a good performance in the range of 90–95 points; less than 90 points is not sufficient to advance to a higher level of the exercise. The obtained results indicate that cognitive-motor training in VR with the posturographic platform is a very sensitive and promising tool for recognizing/assessing functional asymmetries of the right-left side of the body, not only in impaired lateralization but also in training sessions of healthy subjects. The primary application of the VR NEUROFORMA system is the role as an innovative and effective therapeutic method that can support conventional (neuro) therapy and/or (neuro) rehabilitation, as demonstrated by Stryla and Banas’ study in ischemic stroke patients [62]. The researchers reported that the VR environment has a motivating effect on patients and allows them to combine an enjoyable time with effective therapeutic exercises/rehabilitation programs. This computer system (similar to others) offers a variety of interesting virtual exercises combining cognitive and motor tasks in a unique dual-task paradigm with the creation of user training in virtual reality, which supports the process of physical and cognitive training/therapy with dynamic biofeedback in the form of attractive serious mini-games (Figure 3).

Current VR applications indicate an important auxiliary role in improving motor functions, such as joint mobility, muscle strength and endurance, hand-eye coordination, synchronization and control of movement, reaction speed, coordination of opposing movements or balance training with load distribution. Additionally, among the improvements in cognitive functions are effective assistance in focusing attention, inhibiting reflex reactions, exercising memory, improving knowledge management, supporting visual perception, problem-solving, counting and reading, and decision-making processes. In addition, new studies are already emerging showing the beneficial therapeutic effects of not only the classical VR approach (Table 1), but also the more effective use of mirror VR sessions/training, which are based on the phenomenon of mirror neuron plasticity [94]. Examples of various proposed virtual tasks/exercises that can impact cognitive and motor functions used in the upper limbs are shown in Figure 3, and balance control training in Figure 2.

To sum up, new digital technologies are a real hope in view of the prevalence of civilization diseases and the rapid aging of modern societies. As already highlighted, an undoubted benefit of virtual technologies is that they can be used in the home environment. This is particularly evident when opportunities for movement or direct contact with specialists/therapists are limited, e.g., such as during the lockdown caused by the COVID-19 pandemic. Many researchers point to the need to test and validate innovative virtual reality environments and tasks for use in basic and clinical neuroscience [75,95], and thus to formulate recommendations for the design and implementation of VR in clinical screening, diagnosis and therapeutic rehabilitation [96,97,98,99,100]. It should be noted that virtual reality-based assessment can be a good alternative to classical or computer-based neuropsychological assessment due to its greater ecological validity [67,76,101,102]. The basis of physiotherapy is systematic physical exercises adapted to both the severity of the disease and the patient’s effort tolerance. Note that the virtual reality environment allows for safe and systematic exercises, taking into account the capabilities of both healthy individuals and patients. Hence, medical versions of virtual reality are dedicated to a very wide range of users, including different groups of patients. There are a great number of VR interfaces and systems that can be helpful in hospitals, rehabilitation centers and other institutions, providing therapy, rehabilitation or health promotion and disease prevention, especially for the elderly, as shown in Table 2 [18,20,21,22,23,47,103]. Current evidence of the advantages of virtual rehabilitation over traditional exercise is still insufficient, but such training is already a valuable complement to conventional therapy and rehabilitation. Of course, medical applications are of particular importance, raising great hopes in clinical neuroscience, because innovative technologies have huge potential for the unlimited simulation of various situations, individually selected both in terms of exercise/training and the capabilities of each participant.

## 4. Current Limitations and Disadvantages of Virtual Worlds and Their Yet-Unknown Future

As depicted, virtual reality is steadily gaining popularity, not only in gaming, entertainment and business, but also in education, (neuro)science, public health, medical practice and various other fields as well. While it is an innovative, promising technology, one cannot deny the disadvantages of VR, especially regarding the user’s health. For example, a very important issue for the application of VR methods is not only the effective diagnosis of the conditions of examining subjects and beneficial rehabilitation of patients with deficits/disabilities, but also research into the safety and prevention of adverse effects of VR use [104,105,106,107,108,109,110,111] or the treatment of diagnosed/recognized internet gaming disorders [112,113,114,115]. Moreover, the method of interaction is extremely important in these systems, regarding (a) the more capabilities an interface has, (b) the more advanced tasks can be performed with it, (c) interfaces that use the senses, primarily sight, less frequently hearing, voice, touch or taste. State-of-the-art methods of user interaction with the virtual environment (human–computer interaction, HCI) include the use of the latest generation of sensors that recognize and read facial expressions, such as specialized Vive Facial Trackers. The data acquired from this type of sensor can be used to try to read the user’s emotions during exercise [116]. This is an interesting new approach, as emotion recognition involves the perceptual abilities to decode and assign meaning to emotional expressions taking into account both facial expressions and prosodic or postural signals [117,118,119].

It is worth noting that, because perception, vision, and vestibular information are constantly collected and analyzed by the brain, the sense of presence and levels of immersion in VR may cause the user to experience oculomotor or disorientation symptoms, nausea, malaise, and discomfort referred to as cybersickness, which also affects the cognitive and motor skills of VR users (Table 3). Reports suggest that the symptoms are provocative of visual, not-visual or multi-sensory origin and could persist for hours, and in some cases days, after exposure to VR environments. Research on cybersickness has shown that this cybersickness problem is due not only problems with current hardware and software, which are likely to be resolved in the future, but also to a lack of understanding of how cybersickness manifests (several hypotheses include sensory conflicts, neural mismatches, effects of evolutionary origin, ecological influences such as postural instability, or multisensory reweighting such as visuo-vestibular mismatches in VR). In addition, symptoms vary across individuals and potential mitigation strategies are still being intensively explored [100,104,106,107,120]. For example, two primary tools measure cybersickness, such as the Simulator Sickness Questionnaire (SSQ) and the Virtual Reality Sickness Questionnaire (VRSQ). Adverse symptoms of simulator sickness and motion sickness are rare and less intense than symptoms of VR cybersickness. Recently, Kourtesis and co-workers [104] presented an interesting novel Cybersickness in VR Questionnaire (CSQ-VR), which is an improved version of the VRISE (Virtual Reality Induced Symptoms and Effects) section of the VRNQ questionnaire (Virtual Reality Neuroscience Questionnaire). The authors recommend the CSQ-VR as valid for measuring the presence and intensity of VR cybersickness, and suggest that pupil size assessment may be a predictor and potential biomarker of cybersickness intensity. In general, researchers indicate that prolonged use of VR interfaces may cause feelings of fatigue and even symptoms of cybersickness [121,122,123,124]. Some studies demonstrate that non-immersive environments can reduce the risk of cybersickness.

However, recent immersive VR studies showed that cybersickness may be significantly mitigated or avoided. For example, the Kourtesis team [66,67,95,100] proposed the novel immersive VR-EAL environment using the HCT Vive HMD, which fulfills key NAN and AACN criteria, such as safety and effectiveness, end-user identity, technical hardware and software features, privacy and data security, use of reporting services, and reliability of responses and results, and additionally does not cause cybersickness. The researchers demonstrated that on all VRISE items, with the exception of fatigue, there were no adverse symptoms. VR-EAL participants reported only very mild fatigue, although this was an expected outcome as the duration of the VR-EAL tasks was 60 min. Similar fatigue was observed during an 80 min paper-and-pencil session [66]. Zając-Lamparska’s group using the GRANDYS game with Oculus and a control pad [65] in older adults without and with mild dementia identified important exclusion criteria, such as the presence of mental disorders and serious somatic diseases, as well as the presence of visual, auditory and motor impairments that may prevent the use of the game. Patients with MCI may have difficulty understanding and coordinating the game’s control interface. In addition, Oculus prevented participants from visually verifying the selection of buttons on the pad, so they had to maintain not only functional but also spatial mapping in working memory at all times. Similarly, Brachman’s team [70] emphasized that their balance games for healthy elderly people were tailored to the individual abilities of the subject. Among the exclusion criteria were disorders affecting postural stability (e.g., orthopedic or vestibular diseases), as well as diagnosed dementia or vision deficits. Of course, a physiotherapist ensured safety during training and exercise, and participants are asked to report any indisposition or side effects. Buxbaum’s team [60], using the VRLAT test, reported that patients were included if vision, comprehension, attention, and motor skills were sufficient to complete study tasks. However, among the 70 participants, 64 subjects were able to complete all six VRLAT conditions but six patients were unable to complete the participant-driven conditions due to difficulty in operating the joystick.

The effects of practice and fatigue on VR exercise/training performance are valid issues. Research indicates that participants obtained a worse score in early conditions than later, which is consistent with possible subtle practice such as learning effect. Virtual technologies immerse the participant in the surroundings and stimulate their senses to feel as if they are within the virtual experience, changing their perspective for better/different and extraordinary experiences. Still, the continuous development of IT/ICT technologies mixes and breaks down the barriers and limitations of current technologies, creating new and unknown opportunities for technological innovation, for example the prospect of advancing personalization concepts in the immersive world of the metaverse, including future cybersecurity protocols for users [125,126,127]. This perspective of the digital meta-future will be the next-generation internet after the web and mobile versions, and will integrate a variety of emerging technologies including the current digital twin, i.e., a physical and mirror image of the real world (first phase), technology of XR:VR/AR/MR providing an immersive 3D experience (second phase), and then there is the metaverse as the new future world(s) (third phases of development of digital technologies), which is almost surrealistic, i.e., physical and digital worlds are immersed in each other [127,128].

Summarizing, it should be emphasized once again that there are no general recommendations for the use of these innovative IT/ICT technologies in scientific research and biomedicine. The lack of standardization of virtual environments leads to difficulties in replication across studies. Consequently, the variability between assessments makes them incomparable and less reliable. Additionally, it is worth noting that the very nature of both non-immersive and immersive VR largely depends on the use of vision to navigate and perform virtual tests and tasks. Therefore, the inclusion criteria for participants in many studies include normal vision. This can be a limitation for visually impaired patients and the elderly. VR training also requires a certain level of cognitive functioning and frequent use of manually operated real/virtual devices. Equally importantly, the final success depends largely on the motivation of participants to complete VR tasks and/or programs.

Apart from this, there are open questions related to ethics and security in virtual worlds [129,130,131,132], as well as the various costs of these digital technologies [133,134,135,136,137]. The most advanced ones are still very expensive and available in research and medical centers or other institutions. Despite several limitations and the lack of standard protocols for using the VR systems on offer, the data obtained from research to date indicate their usefulness in many areas of human activity. Therefore, modeling studies on healthy individuals are very important for their own safety, as well as for the safety of current and future users in general, especially for the patient population, for improving their health, well-being and coping with daily and occupational activities.

## 5. Conclusions

This review aimed to present interesting findings regarding the effects of virtual tasks and exercise, and virtual cognitive-motor training on executive and motor functions. Despite the lack of valid standards for virtual worlds, data to date from basic and clinical neuroscience, medical practice and other sciences indicate their usefulness and objectivity in quantitatively assessing a wide spectrum of (neuro) physiological, (neuro) psychological, and other bodily functions. In particular, current research on the impact of virtual cognitive and motor exercises on brain health has shown that virtual environments: (a) are very attractive and stimulate the rapid development of modern civilization and the exploration of the capabilities of the human brain; (b) are promising, motivating, easy to individualize and control, and relatively safe for the rebuilding/remodeling motor and cognitive functions in brain disorders; (c) are very effective in supporting the integration of executive and motor functions in dual-task exercises/training. Furthermore, (d) concepts such as the metaverse lead to the consideration of current practices and areas for future research.

Future perspective: tt is not yet known what impact exercise and being in digital worlds may have on brain functions and health, and this still requires further and in-depth interdisciplinary research into basic, clinical or digital neuroscience and other fields of knowledge.

## Figures and Tables

**Table 1:** The examples of multidisciplinary research examining the effects of VR cognitive and motor task/exercise/procedural training in healthy and various patient groups (children, adults, and elderly people). These studies demonstrate the effectiveness of virtual reality methods in assessing brain functions both in health and disease. Moreover, VR tasks can enhance specificity and ecological validity by presenting the patients with functional situations that resemble real daily life.

Authors	Virtual Scenarios, Tasks and Exercises	Basic Purposes of Studies
Buxbaum et al. 2012 [60]	The Virtual Reality Lateralized Attention Test (VRLAT). It was performed for two different navigation tasks: (a) virtual VRLAT test by naming objects presented beside a virtual winding path using a joystick or experimenter’s help (without joystick); and (b) real tests of sensory and motor function and paper-and-pencil (neglect) tests; the VRLAT test contains three levels of array complexity; the testing protocol requires about 90 min.	The study investigates the using the VRLAT task/training in the diagnosis and therapy/rehabilitation of patients with unilateral spatial neglect (USN); this VRLAT is recommended as a sensitive, valid and reliable measure of hemispatial neglect, and as useful for both clinical and research purposes for not only stroke but also dementia or natural aging processes.
Allain et al. 2014 [61]	The non-immersive Virtual Coffee Task (NI-VCT) using a virtual coffee machine: VR environment (on computer screen) was created with Virtools Dev software and was explored with the mouse; participants were instructed to prepare a cup of coffee with milk and sugar in real (RCT) and virtual (NI-VCT) conditions.	The proposal of Virtual Reality Kitchen test (including visual and auditory events) for detecting deficits in Alzheimer’s disease (AD); the NI-VCT might reflect everyday action deficits in AD patients more accurately than other neuropsychological informant-rated measures of everyday function.
Stryla and Banas 2015 [62]	The training program in a non-immersive VR NEUROFORMA environment, which uses motion capture technology with cameras to track and analyze the movement of objects and applying dynamic biofeedback; the participants stand/sit in front of the screen and see themself in the vicinity of VR objects.	To analyze the impact of physiotherapy including VR training on improvement in motor performance of the paresis upper limb in patients after ischemic stroke; and also to determine the effect of VR exercise on non-paresis limb.
Negut et al. 2017 [63]	The Virtual Classroom (VC) tasks; each participant was tested in VR using HMD with and without distractors to assess the impact of distractors on task performance; also, subjects completed traditional neuropsychological paper-and-pencil type tests; the testing session lasted for approximately two h.	The proposal of cognitive diagnosis of children with attention-deficit hyperactivity disorder (ADHD); study shows that virtual classroom test can discriminate between children with ADHD and typically developing (TD) children.
Aubin et al. 2018 [64]	The Virtual Action Planning-Supermarket (VAP-S): all participants met twice: (a) in the non-immersive VAP-S (by interfaces such as screen, mouse and keyboard), and (b) one week after the virtual task, in the real shop, and subjects given the same instructions as for the VR task.	The performance of the shopping task was tested in both virtual and natural (grocery shopping) environments; i.e., the example of the cognitive diagnosis of patients with mild cognitive impairment (MCI), schizophrenia, after stroke or Parkinson’s disease.
Zając-Lamparska et al. 2019 [65]	The eight sessions (two per week) of VR-based cognitive training using the GRADYS game with the Oculus Rift DK2 and Vbox 6DOF control pad; a single session lasted from 45 min to an hour.	The assessment of the efficacy of VR-based cognitive training in relation to cognitive aging both in health and disease, i.e., healthy older versus older with mild cognitive impairment (MCI).
Kourtesis et al. 2020 [66], 2021 [67]	The realistic immersive Virtual Reality Everyday Assessment Lab (VR-EAL) (including real-life situations) and paper-and-pencil testing sessions; VR interfaces: HTC Vive HMD with two lighthouse stations for motion tracking and two HTC Vive wands with six degrees of freedom (6DoF) for navigation and interactions within the virtual environment; the VR scenario lasted 60–70 min.	The assessment of the immersive VR methods in relation to the criteria of the National Academy of Neuropsychology (NAN) and American Academy of Clinical Neuropsychology (AACN); the studies show that the VR-EAL is a safe and enjoyable system without adverse effects and meets the NAN and AACN criteria.
Sokołowska 2021 [68,69]	Sessions of cognitive and motor exercises at three difficulty levels, easy, medium and difficult, with distractors (for the dual-task paradigm) in the non-immersive VR NEUROFORMA with platform; a single session lasted about an hour.	Modeling studies on functional brain lateralization in healthy adults; the assessment of the effectiveness of recognizing functional/postural asymmetries by the non-immersive VR tool.
Brachman et al. 2021 [70]	The balance-based exergaming training in non-immersive (avatar) VR with seven games; this VR system included two integrated different devices such as a force platform and a 3D measurement by Kinect sensor system; training program: 12 sessions, three times a week, 30 min per session.	The evaluation of effects of VR training on balance in healthy elderly women by three tests: quiet standing, functional balance (FBT), and limit of stability (LOS) tests; after a four-week program, significant improvement in LOS performance was observed.
Rutkowski et al. 2021 [71]	Music is a powerful stimulation of the brain, so the training used a commercial immersive VR system in musicians.	The evaluation of (positive) effects of VR training on hand-eye coordination and reaction time in young musicians.
Msika et al. 2022 [72]	The original new REALSoCog task in non-immersive (avatar) VR: participants were asked to navigate in a simulated city environment (computer screen and using keyboard arrow keys) and judge 27 encountered situations that investigated social norms by displaying control/conventional/moral versus transgressions; the VR procedure lasted approximately an hour and a quarter.	Exploratory research in VR focus on the integrative social cognition assessment in normal aging; the example of a new neuropsychology study on social and moral cognition (including socio-cognitive processes such as the ToM or empathy), and showing interesting fields of healthy brain functions (social brain, emotional brain) in natural aging.
Bendixen et al. 2023 [73]	The effects of virtual reality immersion on postural stability during dynamic transition tasks versus traditional measures.	The evaluation of the impact of the VR unanticipated perceptual sport perturbation on postural stability.
De Lorenzis et al. 2023 [74]	Experimental modeling for different Virtual Realty Training Systems (VRTS) regarding issuing learning paths in (neuro) pedagogy.	The evaluation of effects of procedural training and learning paths such as traditional learning (TL) and learning by teaching (LBT).

**Table 2:** The summary of VR applications and equipping virtual environments used in basic and clinical neuroscience.

Basic Issues	Examples and Conditions
Diagnosis	Neurology; neuropsychology; neuropsychiatry
Therapy	Physiotherapy; neurotherapy; mirror therapy
Rehabilitation	Neurological/neuropsychological/post-traumatic rehabilitation; (mirror) neurorehabilitation
Health promotion and disease prevention	Cognitive/motor/balance control training; supporting the development of children with disabilities
Geriatrics	Healthcare of seniors; cognitive-motor exercises and balance control training to improve mobility, prevent falls and train cognitive abilities
Cognitive tasks and exercises	Attention; memory; counting and reading; solving problems; using knowledge; decision making; visual perception; inhibition of reflex reactions
Motor tasks and exercises	Visual-motor coordination, synchronization and motion control; bilateral coordination, reaction speed, muscle strength and endurance; joint mobility, balance training, and load distribution
VR environments with varying degrees of immersion	Non-immersion; extended reality; full immersion
Interfaces using the senses, primarily sight, less so hearing, voice, touch or taste	Keyboard; mouse; joystick; computer monitor; camera (internet camera, Microsoft Kinect controller); special goggles VR/AR/MR (Microsoft HoloLens, Vive Pro Eye); head-mounted displays HMDs (Oculus Rift, HTC Vive); mobile devices (smartphone, tablet); console and controllers (Oculus Touch), Xbox Gamepad controller (Microsoft Xbox Controller) or Leap Motion, Dexmo; gloves; voice commands (Microsoft Azure); facial recognition sensors (Vive Facial Tracker); posturography or treadmill platform; CAVE (cave automatic virtual environment); exoskeleton
Users	Healthy and patient populations (children, adults, the elderly)
User activities	Standing, sitting, lying down position; free movement; ability to choose a preferred range of motion; as a mirror image of a user, avatar, or other virtual object
Type of training	Cognitive/motor/cognitive-motor (single or dual task) training with biofeedback; multi-modal training
Observation and/or recording sessions	3D optical system for precise observation of patients’ activity; remote monitoring and control of training sessions; possibility of recording for offline analysis and evaluation
Additional conditions	Before and during the exercise, adjust the set parameters of the task to the individual needs of each user Progress reports and/or tracking results/effects The ability to apply or continue rehabilitation at home is also an important part of therapy (because exercises of too low frequency and short duration are associated with poorer functional outcomes for improved patients) Sometimes cybersickness symptoms are observed, but mainly associated with immersive VR and after a long time of exposure

**Table 3:** The limitations/disadvantages of VR with regard to brain health.

Disadvantages	Description of Impact of VR on Human Health
Headaches	Overexposure to VR can lead to increased alterations such as headaches and nausea or malaise, dizziness, stomach awareness, and blurred vision
Motion sickness	Distances in VR are often very close, and as a result can disturb the relationship between visual perception and brain processes, leading to feelings of motion sickness
Near-sightedness	VR headsets are placed too close to the eyes, causing strain; overuse can also affect the growth of the eye, leading to myopia or near-sightedness
Hearing impairment/loss	Similarly, overexposure to the high volume at such a close range may have long-term effects on the user’s hearing abilities
Delusions	The virtual and real worlds overlap in the brain, causing confusion and making the user lose touch with what is real and what is not
Postural instability	Postural instability predicts cybersickness susceptibility in HMD users

## Data Availability

Not applicable.
